# Click chemistry based synthesis, cytotoxic activity and molecular docking of novel triazole-thienopyrimidine hybrid glycosides targeting EGFR

**DOI:** 10.1080/14756366.2020.1871335

**Published:** 2021-01-28

**Authors:** Reham R. Khattab, Asma K. Alshamari, Allam A. Hassan, Hussein H. Elganzory, Wael A. El-Sayed, Hanem M. Awad, Eman S. Nossier, Nasser A. Hassan

**Affiliations:** a Photochemistry Department (Synthetic Unit), National Research Centre, Cairo, Egypt; b Chemistry Department, College of Science, Ha'il University, Ha'il, KSA; c Chemistry Department, Faculty of Science, Suez University, Suez, Egypt; d Department of Chemistry, College of Science, Shaqra University, Shaqra, KSA; e Department of Chemistry, College of Science, Qassim University, Buraydah, KSA; f Tanning Materials and Leather Technology Department, National Research Centre, Cairo, Egypt; g Pharmaceutical Medicinal Chemistry, Faculty of Pharmacy (Girls), Al-Azhar University, Cairo, Egypt; h Pharmaceutical Chemistry Department, College of Pharmacy, Shaqra University, Shaqra, KSA

**Keywords:** Click chemistry, thienopyrimidines, glycosides, anticancer, EGFR

## Abstract

In the current study, new thienopyrimidine conjugates bearing 1,2,3-triazole core and different sugar moieties have been designed and synthesized by Cu(I)-catalysed click dipolar cycloaddition. The cytotoxic activity of the synthesised conjugates **2**, **5**, **7**, and **13**–**18** was studied against HCT-116 and MCF-7 cell lines by the MTT assay. The triazole glycosides **16** and **18** provided significant cytotoxic activities against HCT-116 cell lines comparable to that of doxorubicin and other studied compounds. The cytotoxic behaviour against MCF-7 exhibited that all the investigated compounds were more potent than doxorubicin. Moreover, all screened targets were evaluated against mutant EGFR kinase type L858R and the results revealed that the acetylated 1,2,3-triazole glycosides **13**–**18** exhibited excellent EGFR inhibitory activity in comparison with gefitinib. Furthermore, molecular modelling studies were performed to investigate the binding affinity of the most active compounds to EGFR enzyme.

## 1. Introduction

**Figure 1.**
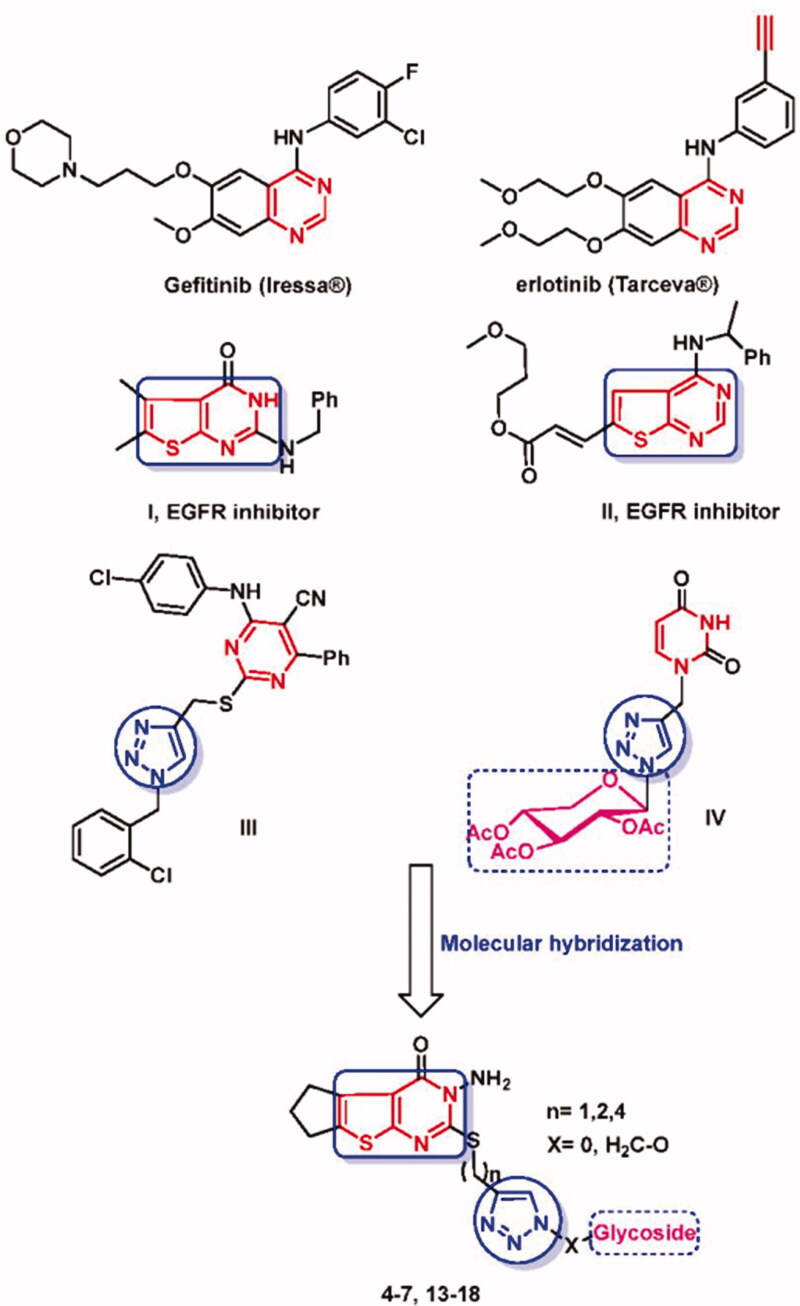
Reported examples of anticancer agents and structural modification to design new thienopyrimidines linked triazole glycosides.

Cancer causes a significant percentage of all human deaths (7.9 million), and it is expected to rise to 12 million deaths per year by 2030^1^. Consequently, discovering new anti-cancer candidates has become a key aim in recent scientific research. Research strategies involving enzyme inhibition pathways are the most followed approaches in cancer drug research. Kinases have been revealed as a group of the most frequent targets of proteins in the field of anticancer drug discovery due to their importance in the regulation of cellular pathways. Research for discovering inhibitors of these targets has found considerable interest owing to their outstanding role in the treatment of cancer and various diseases^2–4^. Among the most widely investigated, receptor tyrosine kinase (RTK) is the epidermal growth factor (EGF) family which is also called erythroblastic B (ErbB) receptors due to its various developmental, physiological functions in human and for triggering cancer^5^. Disruption of the EGFR signalling system, both by antagonistic effects on EGFR binding sites, particularly on the extracellular domain of the receptor or by inactivating intracellular tyrosine kinase, can potentially control (inhibit) the growth EGFR-mediated tumours and show some improvement in the patient's condition. Therefore, EGFR illustrates a valuable role in cancer drug discovery and its inhibitors are now clinically available as gefitinib (Iressa^®^)^6^ and erlotinib (Tarceva^®^)^7^ that have been approved and ratified for the treatment of gastrointestinal stromal tumours and lung cancer (Figure 1).

Based on click strategy, synthetic approaches provide efficient pathways for rapid and mild synthesis of bioactive leads, as possible candidates with enzyme inhibitory actions. The Cu (I)-catalysed 1,3-dipolar cycloaddition of functionalised azide and alkyne substrates which affords 1,4-disubstituted 1,2,3-triazole applying mild conditions exhibits superb selectivity and good yields^8^. One of the most exciting features of click cycloaddition is the unique structural attributes of the yielded 1,2,3-triazole motifs which are known to be present in a large number of compounds owing to their anticancer^9^, and antiviral^10^^,^^11^ activities as well as their ability of being potent pharmacophores^12^^,^^13^. Triazole-amended structures possessing potent anticancer activity were prepared by applying the catalysed azide-alkyne click cycloaddition^14^. Besides, the exceptional plane stability to metabolic biotransformation, the aromatic nature of the triazole core as well as the characterised dipole moment and H-bonding formation, gained the triazole motif more advantages for being a good linker group^15^^,^^16^. On the other hand, incorporation of more than one pharmacophore in one hybrid structure (known as molecular hybridisation) has been found to be an efficient tool for designing new compounds with promising activities^17^. Thieno[2,3-d]pyrimidine system that was afforded through bioisosterism of gefitinib, erlotinib, is an outright ingredient in different cancer chemotherapeutic agents^18–20^ possessing distinct anti-cancer activity via enzymes inhibition mechanism^21–25^. Reported thienopyrimidines, **I** and **II** displayed promising anticancer and kinase inhibitory activity against EGFR^26^^,^^27^ (Figure 1). Furthermore, compounds possessing thienopyrimidine motif were reported with interesting bioactivities such as anticancer^28^^,^^29^, antiviral^30^, antibacterial^31^, and anti-inflammatory^32^ activities. Different glycosyl heterocycles have been synthesised and revealed potential anticancer and enzyme inhibition activities providing essential roles in biological systems, and glycosides possessing the 1,2,3-triazole core, e.g. **III** and **IV**^33–37^ are an essential feature of such compounds (Figure 1).

As a consequence of the above findings, it is assumed that novel hybrid structures possessing the three pharmacophoric systems could be of biological interest. We have continuing research interest for discovering potent and selective anticancer compounds^38^^,^^39^ by the synthesis of new heterocyclic glycosides with modified base and/or glycon constituent. Owing to the significance of the above findings, in the current study, a number of molecular hybrids of thienopyrimidine linked triazole glycosides were synthesised and studied for their anticancer activity in addition to docking studies and EGFR kinase inhibition investigation.

## 2. Results and discussion

### 2.1. Chemistry

**Scheme 1.**
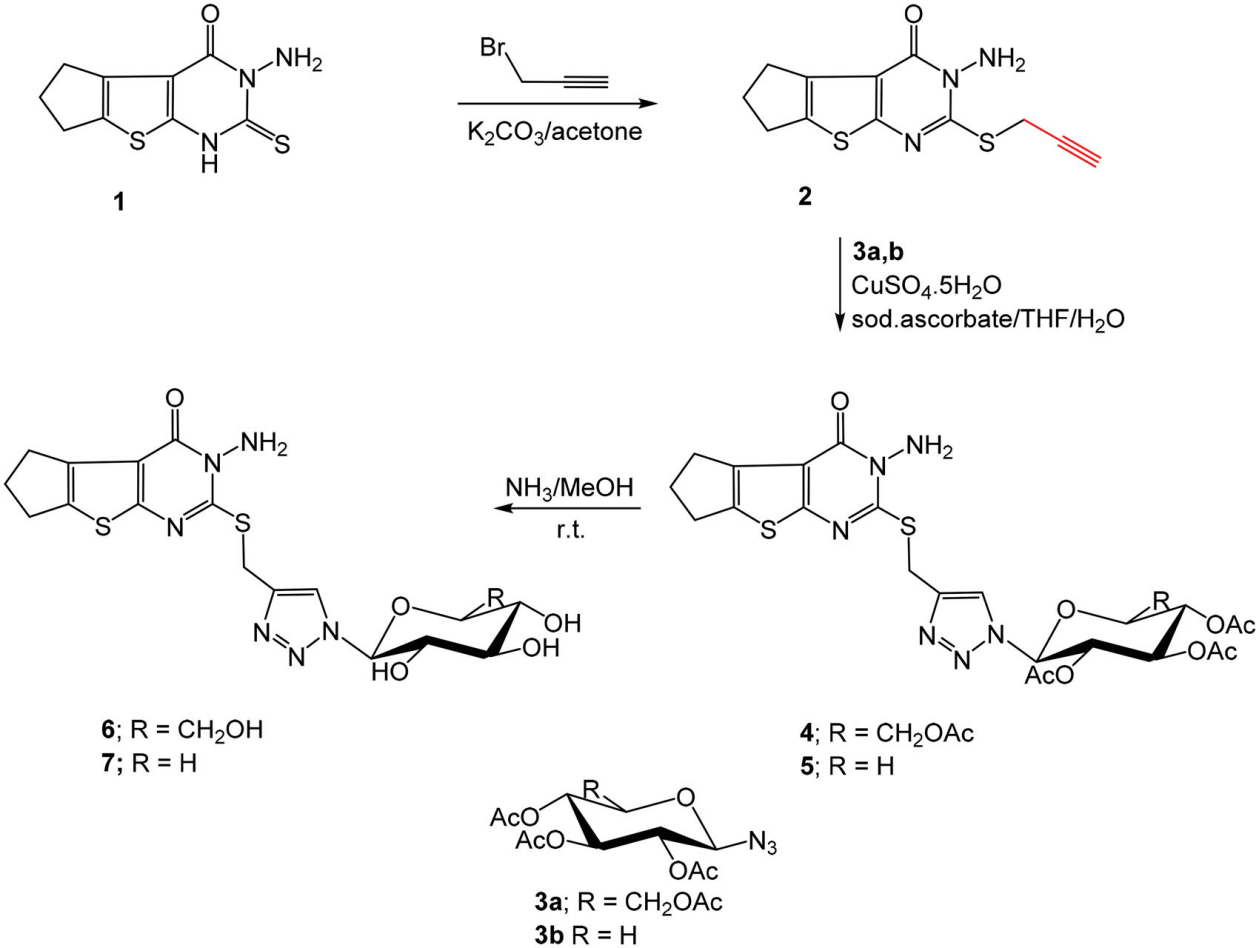
Synthesis of triazole glycosides based thienopyrimidine system.

In the current investigation, two new targeted 1,2,3-triazole linked thienopyrimidine motif glycol-conjugates were synthesised via catalysed click dipolar cycloaddition strategy. The first is a 1,2,3-triazole glycoside in which the carbohydrate moiety is directly attached to the triazole motif via a C-*N*^1^ linkage. The starting acetylenic functionalised thienopyrimidine compound **2** was afforded by reaction of 3-amino-2-thioxothieno[2,3-*d*]pyrimidin-4-one derivative **1**^40^ and propargyl bromide. The terminal acetylenic compound was allowed to react with two glycosyl azides; definitely tetra-*O*-acetyl-*β*-d-gluco- and tri-*O*-acetyl-*β*-d-xylopyranosyl azides (**3a**, **b**), under click dipolar cycloaddition conditions which lead to the formation of the targeted 1,2,3-glycoside derivatives **4** and **5**, respectively in 65–60% yield. Generation of the required Cu (I) catalyst species was performed by means of using sodium ascorbate and copper sulphate, via *in situ* reduction of copper (II) salt. Tetrahydrofuran/water mixture (3:1) was revealed as the best solvent system after studying a number of single and mixed solvent systems (Scheme 1).

**Scheme 2.**
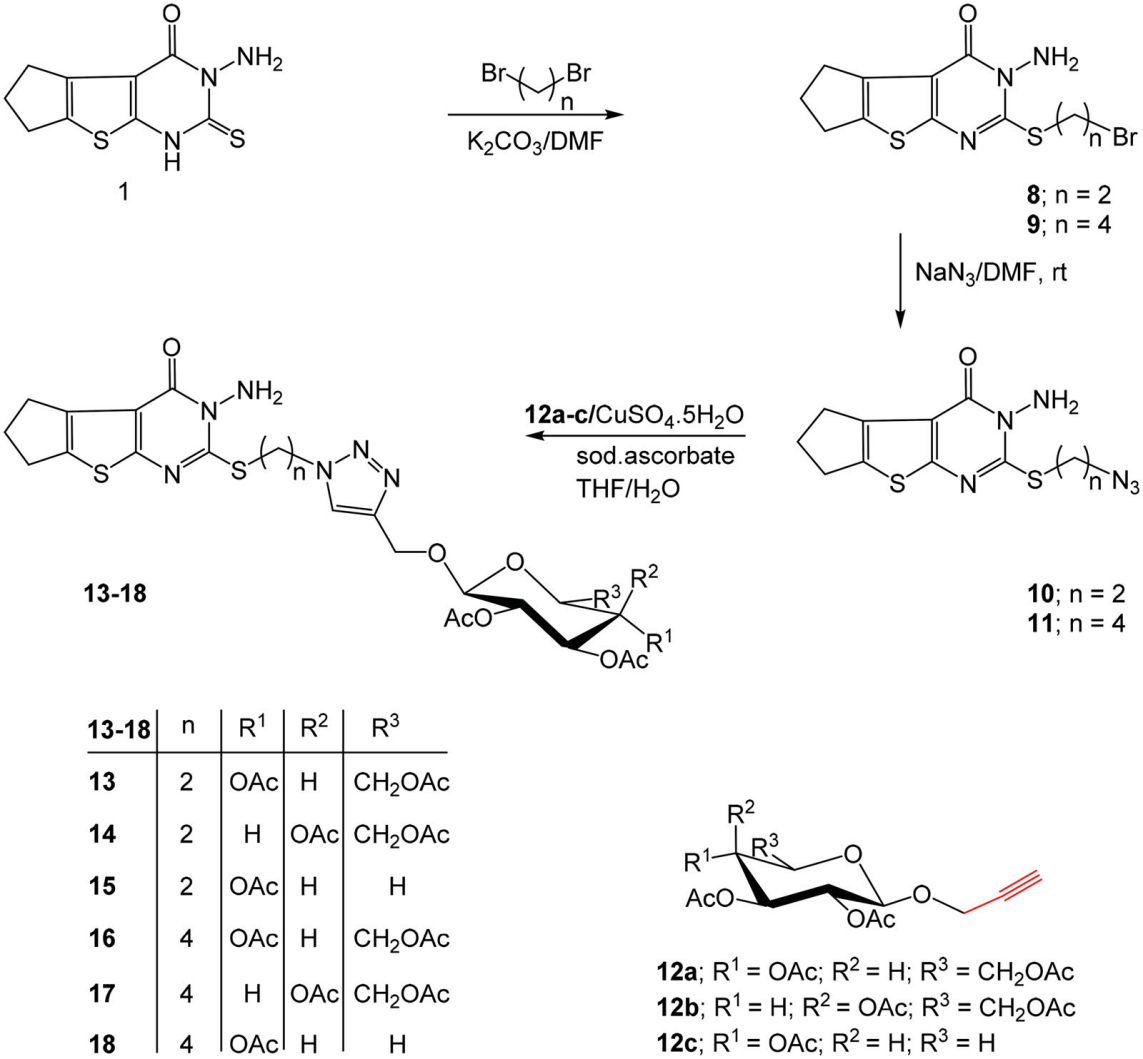
Synthesis of C-linked glycosyl triazoles.

On the other hand, the second thienopyrimidine bridged triazole glycoside derivatives exhibited the 1,2,3-triazole core linked to five and six-carbon sugar moieties since the sugar is attached to the 1,2,3-triazole ring through an oxymethylenic linkage. In such strategy, the azide function was provided into the thienopyrimidine system by halo-alkylation of 3-amino-2-thioxothieno[2,3-*d*]pyrimidin-4-one (**1**) by means of 1,2-dibroalkyl compounds; namely 1,2-dibromoethane or 1,4-dibromobutane, which produced the bromoalkyl derivatives **8** and **9**, respectively in good yield. The latter bromoalkyl compounds were then reacted with sodium azide and afforded the required azide compound **10** and **11**, respectively (Scheme 2).

The formed azide compounds were allowed to react with acetylated acetylenic glucose, galactose, or xylose in a copper catalysed dipolar cycloaddition in THF/H_2_O (3:1) solvent and afforded the targeted glycosyl 1,2,3-triazoles incorporating thienopyrimidine system **13**–**18**.

### 2.2. Cytotoxic activity

The cytotoxic activities were studied *in vitro* against HCT-116 (human colorectal carcinoma) and MCF-7 (human breast adenocarcinoma) cell lines using the MTT assay. The results of the cytotoxicity of the screened compounds against the two carcinoma cell lines were compared to that of doxorubicin used as a reference drug in the current investigation. Their IC_50_ values were calculated and depicted in Table 1. It has been generally observed that most of the tested compounds revealed higher activities against MCF-7 than HCT-116 cell lines. The data shown in Table 1 against HCT-116, indicated that compounds **16**, **17**, and **18** demonstrated the most potent cytotoxic activities (IC_50_=8.1 ± 0.8, 8.4 ± 1.1, and 8.3 ± 1.2 µM, respectively) comparable to that of the reference drug, doxorubicin (IC_50_=7.8 ± 0.7 µM). The remaining derivatives were slightly less active against such cancer cell line (IC_50_ ranged from 8.6 ± 0.9 to 8.9 ± 1.1) compared to the later three compounds. On the other hand, all the investigated synthesised thienopyrimidine compounds were active towards MCF-7 cell lines (IC_50_ ranged from 2.1 ± 0.3 to 5.3 ± 0.8) in comparison with doxorubicin (IC_50_=6.7 ± 0.9 µM). Moreover, the descending order of activity was as follows **2**>**5**>**18**>**13**>**7**>**14**>**15**>**17**>**16**.

**Table 1.** The IC_50_ values of compounds **2**, **5**, **7**, and **13**–**18** against HCT-116 and MCF-7 cancer cells according to the MTT assay.

Compound no.	IC_50_ (µM)±SD	
	HCT-116	MCF-7
**2**	8.6 ± 1.9	2.1 ± 0.3
**5**	8.6 ± 0.9	2.9 ± 0.4
**7**	8.7 ± 1.5	4.3 ± 0.8
**13**	8.7 ± 1.6	4.2 ± 0.5
**14**	8.8 ± 1.1	4.5 ± 0.7
**15**	8.9 ± 1.1	5.0 ± 0.9
**16**	8.1 ± 0.8	5.3 ± 0.8
**17**	8.4 ± 1.1	5.2 ± 0.9
**18**	8.3 ± 1.2	3.9 ± 0.7
**Doxorubicin**	7.8 ± 0.7	6.7 ± 0.9

Correlation of the afforded results with the structural features of the screened derivatives indicated that the attachment of certain glycosyl moieties to the thienopyrimidine ring system resulted in enhanced cytotoxic activities against the HCT-116 cell line and the activity was raised in compounds such as **16**–**18**. For the activity results against MCF-7 cancer cells, the most potent compounds were the terminal acetylenic thienopyrimidine derivative which does not incorporate sugar part. It has also been observed the 1,2,3-triazole glycosides based thienopyrimidine system possessing a linker of four methylene (CH_2_) groups revealed higher activities against HCT-116 cells than their analogues with shorter linker (two CH_2_ groups). Furthermore, it has also been found that the glycosyl-1,2,3-triazole derivatives of the thienopyrimidine system with cyclic glucopyranosyl unit, either acetylated or free hydroxyl moiety, were found more potent against HCT-116 cell line than their analogues with xylopyranosyl fragments. In addition, glycosyl-1,2,3-triazole 18 incorporating the xylopyranosyl moiety showed relatively higher cytotoxic activity against the MCF-7 cancer cell lines more than the gluco- and galactopyranosyl analogues.

### 2.3. Kinase inhibitory activity

The RTKs involving epidermal growth factor receptor (EGFR) and its three related proteins (the ERBB family) have been shown to play essential roles cancerous conditions in addition to their role in normal physiological conditions^41^. The investigated compounds were studied for their inhibitory activity against mutant EGFR kinase type L858R. The resulted IC_50_ values are displayed in Table 2 in comparison with the reference drug, gefitinib (IC_50_=0.014 ± 0.18 µM). According to the revealed results, the acetylated 1,2,3-triazole glycosides **13**–**18** exhibited excellent EGFR inhibition with IC_50_ in the range of 0.010 ± 0.14 to 0.020 ± 0.23 µM. Furthermore, the glycosyl-1,2,3-triazole **18** incorporating the acetylated xylopyranosyl moiety displayed the most potent activity (IC_50_=0.010 ± 0.14 µM) among this series of studied compounds. It is proposed that the direct attachment of the 1,2,3-triazolyl and glycopyranosyl moieties in the synthesised glycosides **5** and **7** resulted in drastic decrease in the inhibitory activity (IC_50_=0.130 ± 1.45 and 0.170 ± 0.43 µM, respectively) due to the absence of the separating linker. Additionally, compound **2** was found to be of weak inhibitory activity of EGFR enzyme (IC_50_=0.500 ± 1.00 µM).

### 2.4. Molecular docking study

Based on the results of kinase inhibitory assessment, we have been promoted to perform a molecular docking study of the studied compounds **2**, **5**, **7**, and **13**–**18** against EGFR kinase by using MOE software version 2008.10. The binding site of EGFR (PDB ID: 3UG2) with its inhibitor gefitinib was retrieved from PDB bank http://www.rcsb.org/pdb. Redocking of the reference ligand showed that the root mean square difference (RMSD) between the top docking pose and original crystallographic geometry of the cocrystallised ligand, gefitinib was 0.87 ˚A with a perfect superimposition between them (Figure 2(C)).

Figure 2.(A) 2D molecular interactions of gefitinib with amino acids of the EGFR enzyme pocket (PDB code: 3UG2). (B) 3D molecular interactions of gefitinib with amino acids of the EGFR enzyme pocket (PDB code: 3UG2). (C) 3D representation of the superimposition of the docking pose (yellow) and the co-crystallised (red) of gefitinib with an RMSD of 0.87 Å.

**Figure:**
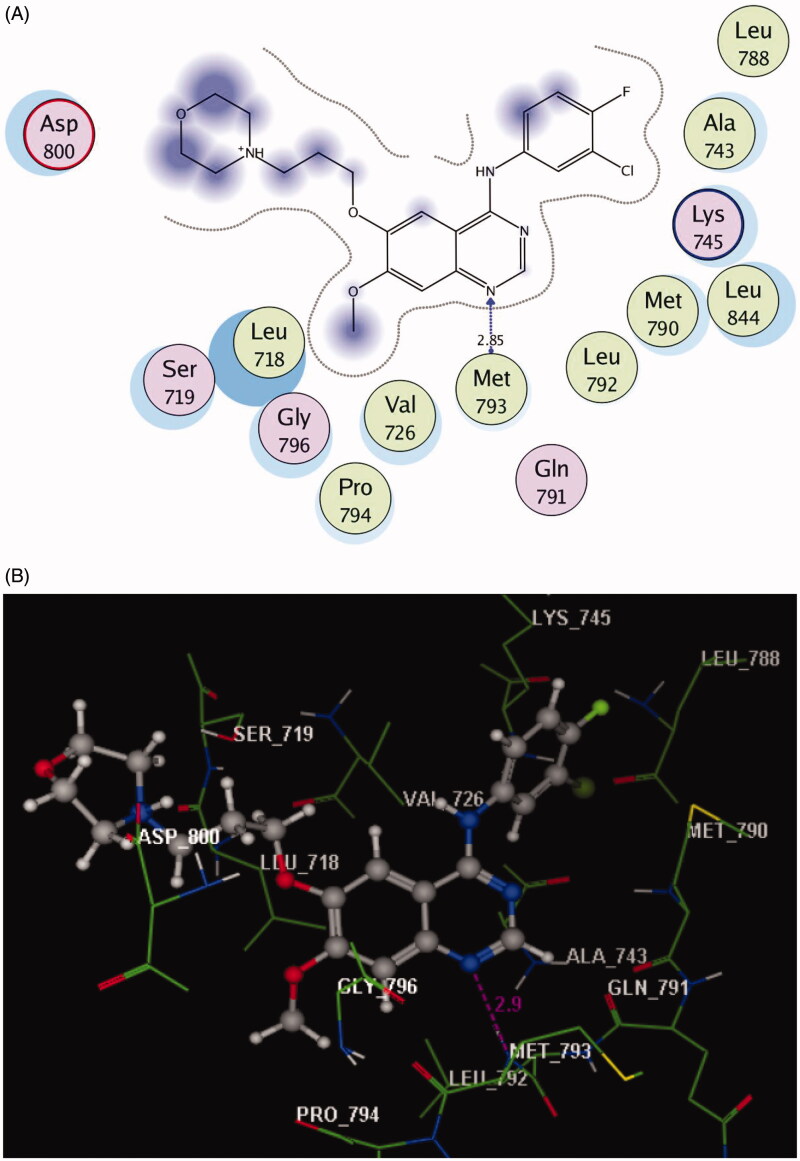

**Figure:**
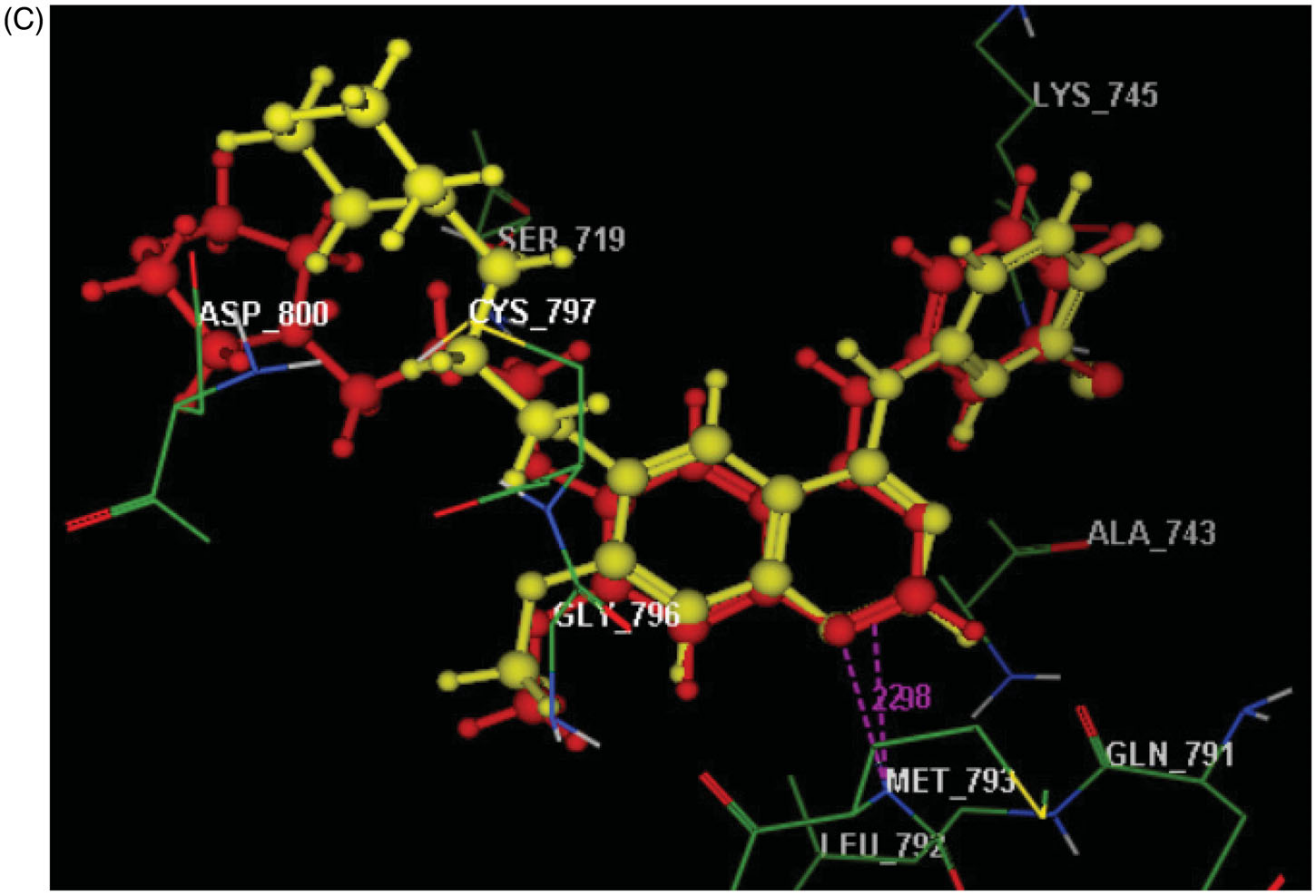

As reported in the docking profile of gefitinib bearing a quinazoline core, the N1 formed hydrogen bond acceptor with the backbone of **Met793** (distance: 2.85 Å) (Figure 2(A,B)). Moreover, the whole structure was inserted nicely within the active site of EGFR through hydrophobic interactions with the essential amino acids **Leu718**, **Ser719**, **Val726**, **Ala743**, **Lys745**, **Leu788**, **Met790**, **Gln791**, **Leu792**, **Pro794**, **Gly796**, **Asp800**, and **Leu844**^42^.

The data of docking scores (kcal/mol) and interactive binding residues of the studied compounds **2**, **5**, **7**, and **13**–**18** are depicted in Table 3. It can be noticed that all derivatives displayed variable and promising energy scores ranging from −7.30 to −9.20 kcal/mol and bind to the key amino acid **Met793** like the original ligand, gefitinib.

**Table 2.** Inhibitory evaluation of the tested compounds against EGFR_L858R_.

Compound no.	IC_50_ (mean ± SEM) (µM)
	EGFR
**Gefitinib**	0.014 ± 0.18
**2**	0.500 ± 1.00
**5**	0.130 ± 1.45
**7**	0.170 ± 0.43
**13**	0.015 ± 0.81
**14**	0.013 ± 0.22
**15**	0.017 ± 0.30
**16**	0.014 ± 0.12
**17**	0.020 ± 0.23
**18**	0.010 ± 0.14

IC_50_: compound concentration required to inhibit the enzyme activity by 50%; SEM: standard error mean; each value is the mean of three values.

**Figure 3.**
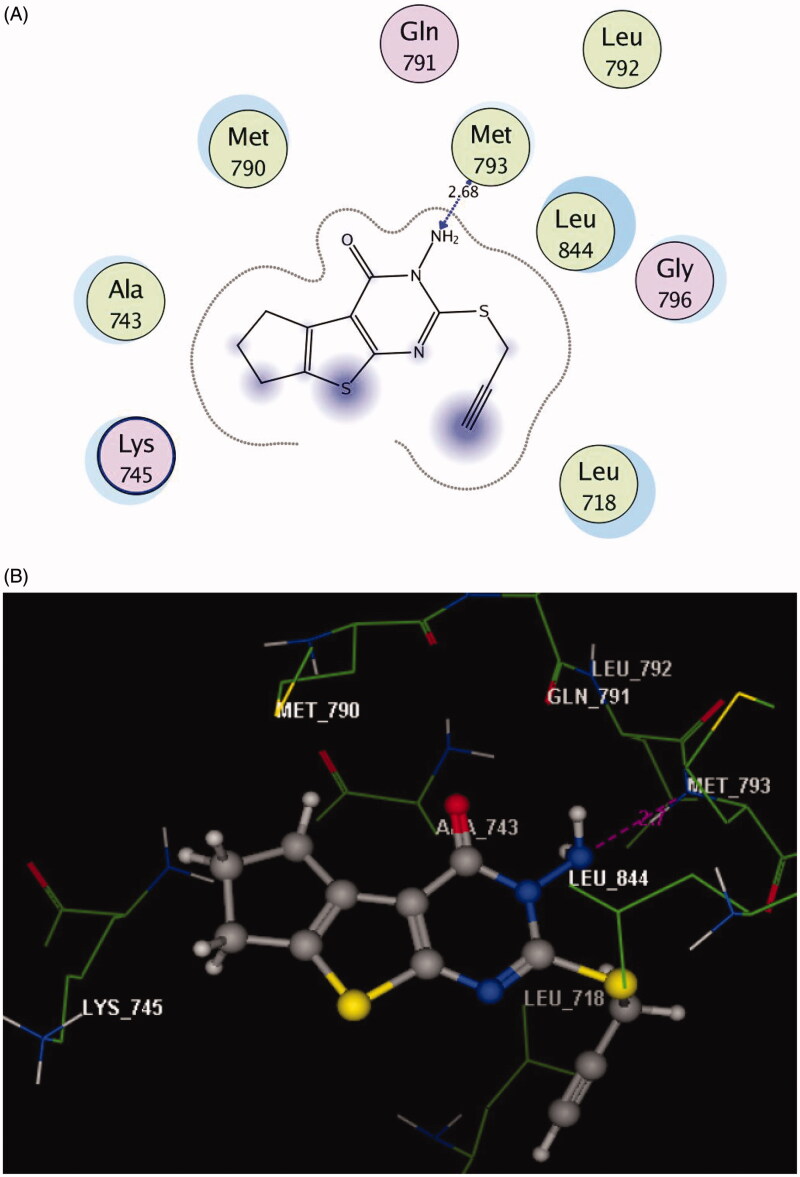
(A) 2D molecular interactions of compound **2** with amino acids of the EGFR enzyme pocket (PDB code: 3UG2). (B) 3D molecular interactions of compound **2** with amino acids of the EGFR enzyme pocket (PDB code: 3UG2).

For example, the lowest biologically active derivative **2** anchored with EGFR binding site via hydrogen bond interaction between the N of the amino group at p-3 of cyclopenta[4,5]thieno[2,3-*d*]pyrimidinone with **Met793** (distance: 2.68 Å) (Figure 3).

**Figure 4.**
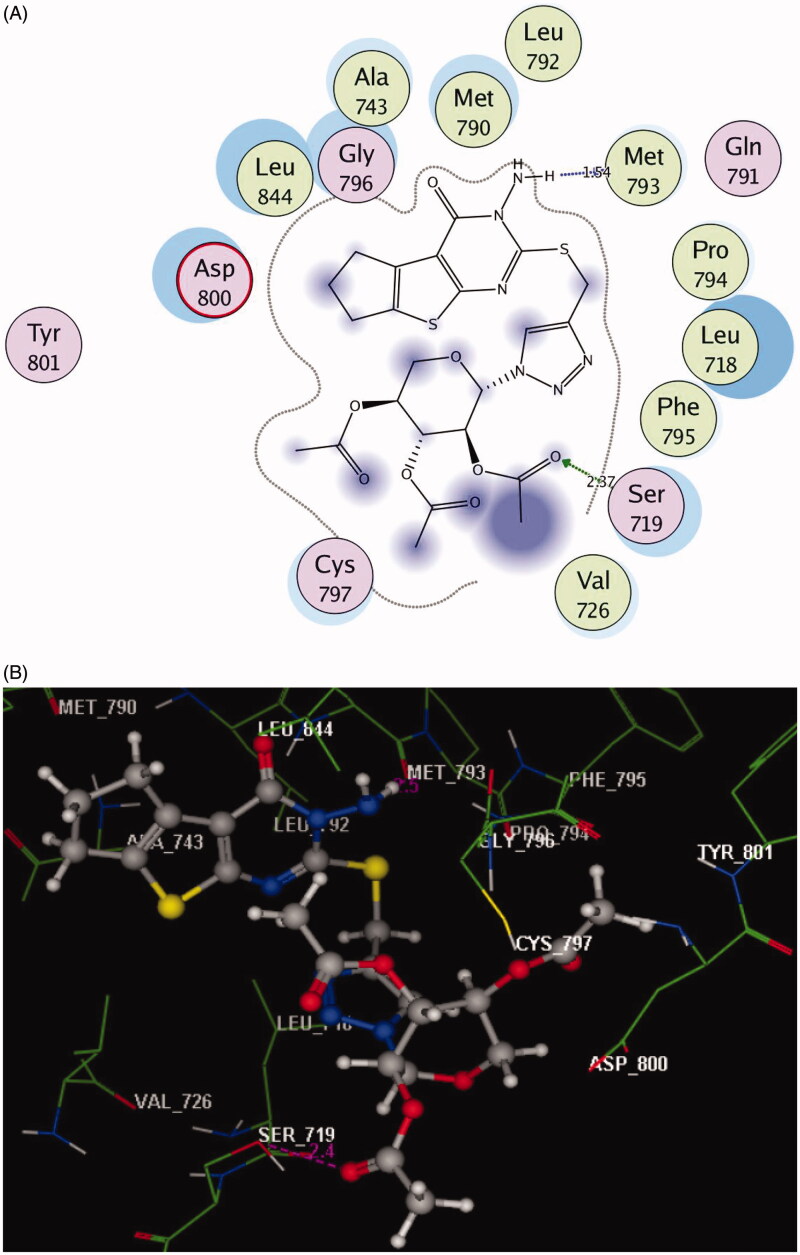
(A) 2D molecular interactions of compound **5** with amino acids of the EGFR enzyme pocket (PDB code: 3UG2). (B) 3D molecular interactions of compound **5** with amino acids of the EGFR enzyme pocket (PDB code: 3UG2).

**Figure 5.**
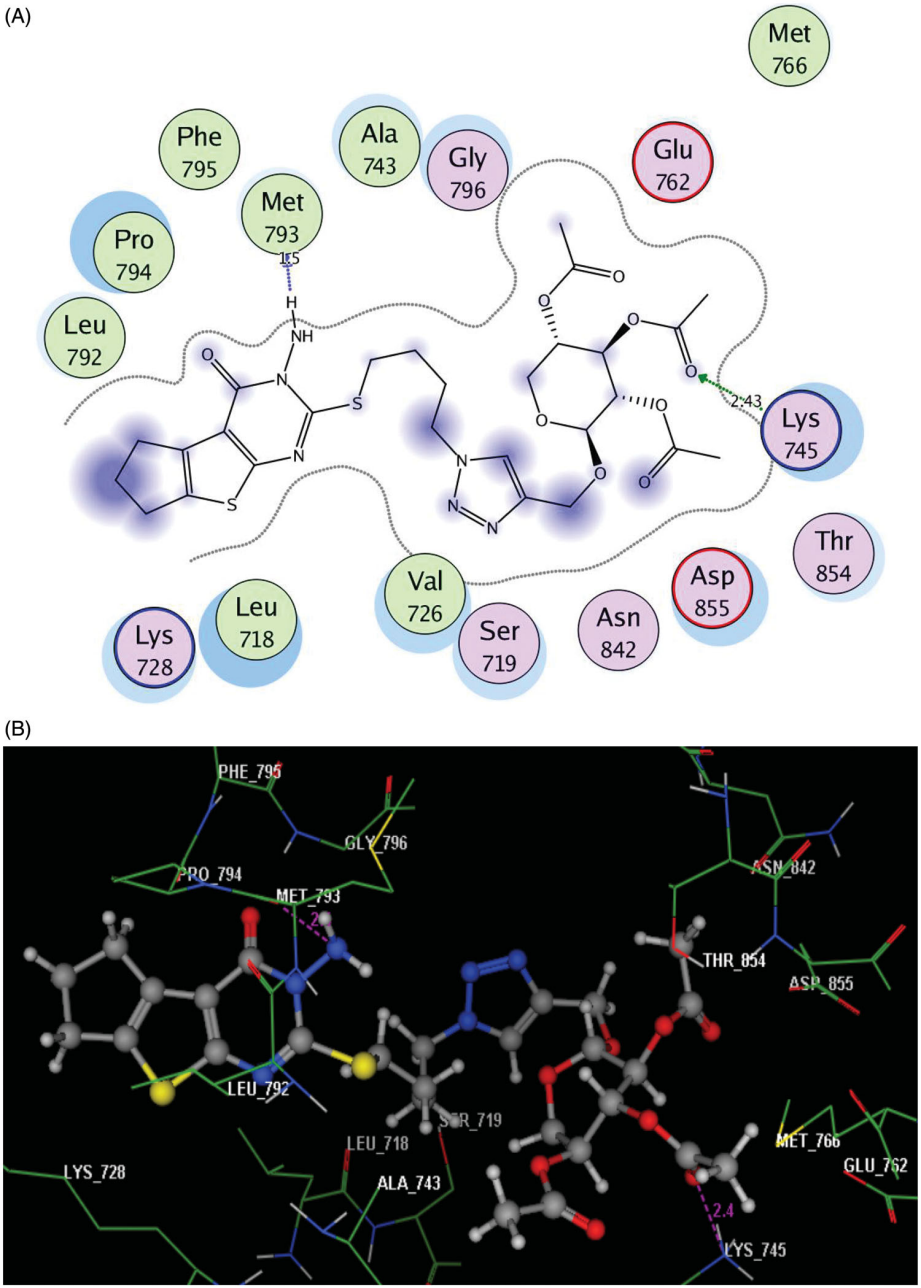
(A) 2D molecular interactions of compound **18** with amino acids of the EGFR enzyme pocket (PDB code: 3UG2). (B) 3D molecular interactions of compound **18** with amino acids of the EGFR enzyme pocket (PDB code: 3UG2).

Incorporation of the 3-amino-2-thioxothieno[2,3-*d*]pyrimidin-4-one nucleus with 1,2,3-triazole and acetylated glycoside scaffolds gave the chance for more fitting within the ATP pocket through different interactions. As observed in Figures 4 and 5, the oxygen of the acetyl group established extra H-bond acceptors with the sidechain of **Ser719** in the triazole-N-glycoside **5** (with moderate inhibitory activity and bearing 1C distance chain) and with the sidechain of **Lys745** in triazole-oxymethyl-glycoside **18** (with the highest inhibitory activity and bearing 4C distance chain) (distance: 2.37 and 2.43 Å, respectively).

Overall, the docking study, along with *in vitro* EGFR results, confirmed that the presence of triazole, oxymethyl, and *β*-d-glycosyl moieties greatly contribute to the chain elongation and the enhancement of the inhibitory activities of these 3-aminocyclopenta[4,5]thieno[2,3-*d*]pyrimidin-4-one-based compounds through contributing in formation of extra significant H-bonds inside the ATP-binding cavity.

## 3. Conclusions

A series of novel 3-aminocyclopenta[4,5]thieno[2,3-*d*]pyrimidin-4-one hybrids linked with 1,2,3-triazol and different glycosides were designed, synthesised and evaluated for their cytotoxic activity against two cancer cell lines HCT-116 and MCF-7 by the MTT assay. Regarding to HCT-116 cell lines, the triazole glycosides **16** and **18** revealed the highest cytotoxic activity in comparison with doxorubicin, while all the studied derivatives displayed excellent activity against MCF-7. The newly synthesised compounds **2**, **5**, **7**, and **13**–**18** were investigated *in vitro* for their suppression activity against mutant EGFR kinase type L858R (Table 3). The acetylated 1,2,3-triazole glycosides **13**–**18** illustrated excellent EGFR inhibitory activity in comparison with gefitinib. Additionally, the molecular docking studies represented the binding orientations of the promising compounds in the active pocket of EGFR and it could be deduced that the 3-aminocyclopenta[4,5]thieno[2,3-*d*]pyrimidin-4-one nucleus attached to acetylated 1,2,3-triazole-oxymethylglycoside moiety improved the binding strength within the active site through H-bonding with the key amino acids **Met793** and **Lys745**. The previous findings may provide promising templates in the anticancer field.

**Table 3.** Docking results of the compounds **2**, **5**, **7**, and **13**–**18** with EGFR kinase (PDB code: 3UG2) using MOE software version 2008.10.

Compound	Docking score (kcal/mol)	Amino acid residues (bond length, Å)	Atoms of compound	Type of bond
**Gefitinib**	–8.15	Met793(2.85)	N1(Quinazoline)	H-acc
**2**	–7.30	Met793(2.68)	N(NH_2_)	H-acc
**5**	–8.22	Met793(1.54);	H(NH_2_);	H-don
		Ser719(2.37)	O(CO)(OCOCH_3_)	H-acc
**7**	–7.62	Met793(1.60);	H(NH_2_);	H-don
		Ser719(2.24)	O(OH)	H-acc
**13**	–8.40	Met793(1.51);	H(NH_2_);	H-don
		Lys745(2.33)	O(CO)(OCOCH_3_)	H-acc
**14**	–8.25	Met793(1.65);	H(NH_2_);	H-don
		Lys745(2.27)	O(CO)(OCOCH_3_)	H-acc
**15**	–7.92	Met793(1.55);	H(NH_2_);	H-don
		Lys745(2.22)	O(CO)(OCOCH_3_)	H-acc
**16**	–8.65	Met793(1.50);	H(NH_2_);	H-don
		Lys745(2.40)	O(CO)(OCOCH_3_)	H-acc
**17**	–8.36	Met793(1.66);	H(NH_2_);	H-don
		Lys745(2.55)	O(CO)(OCOCH_3_)	H-acc
**18**	–9.20	Met793(1.50);	H(NH_2_);	H-don
		Lys745(2.43)	O(CO)(OCOCH_3_)	H-acc

## 4. Experimental

### 4.1. Chemistry

All melting points were measured using a Reichert Thermovar apparatus and are uncorrected. Yields listed are of isolated compounds. The IR spectra were recorded on a Perkin-Elmer model 1720 FTIR spectrometer for KBr disc (Waltham, MA). Routine NMR measurements were made on a Bruker AC-300 or DPX-300 spectrometer (Billerica, MA). Chemical shifts were reported in *δ* scale (ppm) relative to TMS as a reference standard and the coupling constants *J* values are given in Hz. The progress of the reactions was monitored by TLC using aluminium silica gel plates 60 F245. IR, ^1^H NMR, ^13^C NMR, and elemental analyses were performed at the Micro analytical centre at the Faculty of Science, Cairo University, Cairo, Egypt. The starting compound 3-amino-2-thioxo-1,2,3,5,6,7-hexahydro-4*H*-cyclopenta[4,5]thieno[2,3-*d*]pyrimidin-4-one (**1**) was synthesised as previously reported^40^. mp 253–255 °C.

#### 4.1.1. 3-Amino-2-(prop-2-yn-1-ylthio)-3,5,6,7-tetrahydro-4H-cyclopenta[4,5]thieno[2,3-d]pyrimidin-4-one (2)

To a mixture of aminothienopyrimidine derivative **1** (1.195 g, 5 mmol) with propargyl bromide (0.594 g, 5 mmol) and K_2_CO_3_ (0.691 g, 5 mmol) in acetone (15 ml), the reaction mixture was stirred at room temperature for 14 h. The reaction mixture poured in into ice-water; the solid obtained was filtered off, dried and recrystallised from DMF to give the corresponding derivatives **2**.

Yield 75%, mp 181–182 °C, IR (KBr, *υ*, cm^−1^): 3391, 3296 (NH_2_), 2218 (C–C alkyne), 1686 (C═O); ^1^H NMR (DMSO-d_6_, *δ* ppm): 2.38–2.40 (m, 2H, CH_2_), 2.51 (t, 2H, *J*= 1.8 Hz, CH_2_), 2.90 (t, 2H, *J*= 7.2 Hz, CH_2_), 3.29 (s, 1H, CH), 3.83 (s, 2H, CH_2_), 5.78 (br s, 2H, NH_2_ exchangeable with D_2_O). Anal. Calcd. for C_12_H_11_N_3_OS_2_ (277.36): C, 51.97; H, 4.00; N, 15.15. Found: C, 51.80; H, 4.07; N, 15.02.

#### 4.1.2. General procedure synthesis of 1,2,3-triazole acetylated N-glycosides derivatives (4, 5)

To a well stirred solution of the terminal acetylenic derivative **2** (0.554 g, 2.0 mmol) in a mixture of THF–H_2_O (1:2, 15 ml) was added the azido-sugar (2,3,4,6-tetra-*O*-acetyl-d-glucopyranosyl or 2,3,4-tri-*O*-acetyl-d-xylopyranosyl azide) (2.0 mmol). Sodium ascorbate (0.08 g, 0.4 mmol) and few drops of diisopropylethylamine (DIPEA) followed by CuSO_4_·5H_2_O (0.4 mmol, 0.11 g) were then added. The mixture was stirred at room temperature overnight (TLC, petroleum ether–ethyl acetate (4:1)). Extraction of the organic compound layer was performed by shaking the mixture two times for 5 min with ethyl acetate. The organic layers were combined, dried over Na_2_SO_4_ and the solvent evaporated. Purification by column chromatography (hexane/ethyl acetate, 5:1, as the eluent) gave the title products.

#### 4.1.3. 3-Amino-2-(((1-(2,3,4,6-tetra-O-acetyl-β-d-glucopyranosyl)-1H-1,2,3-triazol-4-yl)methyl)thio)-3,5,6,7-tetrahydro-4H-cyclopenta [4,5]thieno[2,3-d]pyrimidin-4-one (4)

Yield: 65%, mp 151–152 °C. IR (KBr, *υ*, cm^−1^): 3440 (NH_2_), 1751 (C═O), 1668 (C═O); ^1^H NMR (DMSO-d_6_, *δ* ppm): 1.94, 1.95, 1.97, 2.01 (4s, 12H, 4CH_3_), 2.27–2.34 (m, 2H, CH_2_), 2.39 (t, 2H, *J* = 1.8 Hz, CH_2_), 2.89 (t, 2H, *J* = 7.2 Hz, CH_2_), 3.82 (s, 2H, CH_2_), 4.05–4.09 (m, 1H, H^5′^), 4.30 (dd, 1H, *J* = 11.2, 3.7 Hz, H^6′^), 4.31 (dd, 1H, *J* = 8.2, 6.4 Hz, H^6″^), 5.14–4.19 (m, 1H, H^4′^), 5.50–5.57 (m, 1H, H^3′^), 5.73 (t, 1H, *J*= 8.2 Hz, H^2′^), 5.77 (br s, 2H, NH_2_ exchangeable with D_2_O), 6.32 (d, 1H, *J*= 8.7 Hz, H^1′^), 8.26 (s, 1H, triazole-H); ^13^C NMR (DMSO-d_6_, *δ* ppm): 20.69, 20.82, 20.89, 26.05, 27.72, 29.06, 29.51, 39.35, 67.98, 68.20, 70.47, 72.56, 73.67, 84.22, 115.99, 123.01, 136.67, 139.42, 144.01, 157.64, 159.42; 166.47, 196.80, 169.98, 170.43, 170.47. Anal. Calcd. for C_26_H_30_N_6_O_10_S_2_ (650.68): C, 47.99; H, 4.65; N, 12.92. Found: C, 47.70; H, 4.51; N, 13.06.

#### 4.1.4. 3-Amino-2-(((1-(2,3,4-tri-O-acetyl-β-d-xyloopyranosyl)-1H-1,2, 3-triazol-4-yl)methyl)thio)-3,5,6,7-tetrahydro-4H-cyclopenta[4,5]thieno[2,3-d]pyrimidin-4-one (5)

Yield: 60%, mp 155–156 °C. IR (KBr, *υ*, cm^−1^): 3432 (NH_2_), 1741 (C═O), 1666 (C═O), ^1^H NMR (DMSO-d_6_*, δ* ppm): 1.97, 1.98, 2.01 (3s, 9H, 3CH_3_), 2.26–2.31 (m, 2H, CH_2_), 2.48 (t, 2H, *J*= 1.8 Hz, CH_2_), 2.88 (t, 2H, *J*= 7.2 Hz, CH_2_), 4.00 (s, 2H, CH_2_), 4.29 (dd, 1H, *J*= 3.7, 10.3 Hz, H^5′^), 4.31 (dd, 1H, *J*= 10.2, 6.8 Hz, H^5″^), 4.97–5.03 (m, 1H, H^4′^), 5.25–5.30 (m, 1H, H^3′^), 5.57 (t, 1H, *J*= 8.2 Hz, H^2′^), 5.73 (br s, 2H, NH_2_ exchangeable with D_2_O), 6.30 (d, 1H, *J*= 8.7 Hz, H^1′^), 8.25 (s, 1H, triazole-H), ^13^C NMR (DMSO-d_6_, *δ* ppm). Anal. Calcd. for C_23_H_26_N_6_O_8_S_2_ (578.62): C, 47.74; H, 4.53; N, 14.52. Found: C, 47.60; H, 4.57; N, 14.46.

#### 4.1.5. General procedure synthesis of 3-amino-2-(((1-(β-d-glycopyranosyl)-1H-1,2,3-triazol-4-yl)methyl)thio)-3,5,6,7-tetrahydro-4H-cyclopenta[4,5]thieno[2,3-d]pyrimidin-4-one (6, 7)

A solution of the acetylated glycoside compound **4** or **5** (0.5 g) in a saturated methanolic ammonia (20 ml) was stirred at room temperature for 7 h. Upon completion of the deacetylation process (TLC, petroleum ether–hexane, 2:1), the solvent was evaporated under reduced pressure at 40 °C to give a residue, which was triturated with diethyl ether (25 ml) to afford a solid which was filtered off, dried and crystallised from ethanol to give **6** or **7**, respectively.

#### 4.1.6. 3-Amino-2-(((β-d-glucopyranosyl)-1H-1,2,3-triazol-4-yl)methyl) thio)-3,5,6,7-tetrahydro-4H-cyclopenta[4,5]thieno[2,3-d]pyrimidin-4-one (6)

Yield: 55%, mp 180–182 °C. IR (KBr, *υ*, cm^−1^): 3450–3421 (OH), 3419 (NH_2_), 1669 (C═O), ^1^H NMR (DMSO-d_6_, *δ* ppm): 2.30–2.38 (m, 2H, CH_2_), 2.47 (t, 2H, *J*= 1.8 Hz, CH_2_), 2.93 (t, 2H, *J*= 7.2 Hz, CH_2_), 4.09 (s, 2H, CH_2_), 4.10–4.19 (m, 2H, H^6′,6″^), 4.27–4.31 (m, 1H, H^5″^), 4.34–4.43 (m, 2H, H^4′,3′^), 4.71–4.75 (m, 1H, OH), 4.97–5.17 (m, 2H, OH. H^2′^), 5.49–5.60 (m, 2H, 2OH), 5.75 (br s, 2H, NH_2_ exchangeable with D_2_O), 5.88 (d, 1H, *J*= 8.2 Hz, H^1′^), 8.36 (s, 1H, triazole-H). Anal. Calcd. for C_18_H_22_N_6_O_6_S_2_ (482.53): C, 44.81; H, 4.60; N, 17.42. Found: C, 44.69; H, 4.66; N, 17.51.

#### 4.1.7. 3-Amino-2-(((1-(β-d-xylopyranosyl)-1H-1,2,3-triazol-4-yl)methyl) thio)-3,5,6,7-tetrahydro-4H-cyclopenta[4,5]thieno[2,3-d]pyrimidin-4-one (7)

Yield: 60%, mp 189–190 °C. IR (KBr, *υ*, cm^−1^): 3448–3419 (OH), 3429 (NH_2_), 1665 (C═O), ^1^H NMR (DMSO-d_6_, *δ* ppm): 2.28–2.36 (m, 2H, CH_2_), 2.50 (t, 2H, *J*= 1.8 Hz, CH_2_), 2.95 (t, 2H, *J*= 7.2 Hz, CH_2_), 4.11 (s, 2H, CH_2_), 4.31–4.38 (m, 2H, H^5′,5″^), 4.39–4.48 (m, 2H, H^4′,3′^), 4.49–4.53 (m, 1H, OH), 4.98–5.07 (m, 2H, OH. H^2′^), 5.43–5.40 (m, 1H, OH), 5.72 (br s, 2H, NH_2_ exchangeable with D_2_O), 5.85 (d, 1H, *J*= 8.2 Hz, H^1′^), 8.34 (s, 1H, triazole-H) Anal. Calcd. for C_17_H_20_N_6_O_5_S_2_ (452.50): C, 45.12; H, 4.46; N, 18.57. Found: C, 44.88; H, 4.51; N, 18.41.

#### 4.1.8. General procedure synthesis of 3-amino-2-((4-bromoalkyll)thio)-3,5,6,7-tetrahydro-4H-cyclopenta[4,5]thieno[2,3-d]pyrimidin-4-one (8, 9)

To a solution of the aminothienopyrimidine derivative **1** (1.195 g, 5 mmol) in DMF (15 ml) was added to anhydrous K_2_CO_3_ (0.691 g, 5 mmol) and the mixture was stirred at room temperature for (1 h). 1,2-Dibromoethane or 1,2-dibromobutane (5 mmol) was slowly added under vigorous stirring, and the resulting mixture was stirred at room temperature for further 10 h. Water (100 ml) was added.

#### 4.1.9. 3-Amino-2-((2-bromoethyl)thio)-3,5,6,7-tetrahydro-4H-cyclopenta[4,5]thieno[2,3-d]pyrimidin-4-one (8)

Yield: 75%, mp 169–170 °C. IR (KBr, *υ*, cm^−1^): 3442 (NH_2_), 1665 (C═O), ^1^H NMR (DMSO-d_6_, *δ* ppm): 2.30–2.38 (m, 2H, CH_2_), 2.59 (t, 2H, *J*= 2.8 Hz, CH_2_), 2.98 (t, 2H, *J*= 7.2 Hz, CH_2_), 3.38 (t, 2H, *J*= 7.2 Hz, CH_2_), 3.65 (t, 2H, *J*= 7.2 Hz, CH_2_), 5.89 (br s, 2H, NH_2_ exchangeable with D_2_O); MS: *m/z* (%)=346 (M+, 20). Anal. Calcd. for C_11_H_12_BrN_3_OS_2_ (346.26): C, 38.16; H, 3.49; N, 12.14. Found: C, 38.05; H, 3.45; N, 12.28.

#### 4.1.10. 3-Amino-2-((4-bromobutyl)thio)-3,5,6,7-tetrahydro-4H-cyclopenta[4,5]thieno[2,3-d]pyrimidin-4-one (9)

Yield 77%, mp 161–162 °C. IR (KBr, *υ*, cm^–1^): 3439 (NH_2_), 1666 (C═O), ^1^H NMR (DMSO-d_6_, *δ* ppm): 2.09–2.18 (m, 2H, CH_2_), 2.42–2.53 (m, 2H, CH_2_), 2.55 (m, 2H, CH_2_), 2.90 (t, 2H, *J*= 7.2 Hz, CH_2_), 2.93 (t, 2H, *J*= 6.9 Hz, CH_2_), 3.17 (t, 2H, *J*= 6.9 Hz, CH_2_), 3.41 (t, 2H, *J*= 6.6 Hz, CH_2_), 5.77 (br s, 2H, NH_2_ exchangeable with D_2_O). Anal. Calcd. for C_13_H_16_BrN_3_OS_2_ (374.32): C, 41.71; H, 4.31; N, 11.23. Found: C, 41.52; H, 4.38; N, 11.14.

#### 4.1.11. General procedure synthesis of 3-amino-2-((azidoethyl)thio)-3,5,6,7-tetrahydro-4H-cyclopenta[4,5]thieno[2,3-d]pyrimidin-4-one (10, 11)

A mixture of bromoalkyl derivatives **8** or **9** (3 mmol) and sodium azide (0.292 g, 4.5 mmol) in DMF (15 ml) was stirred at room temperature for (14 h). The reaction mixture was poured into ice-water; the solid obtained was filtered off, dried and recrystallised from DMF–water (1:1) to give the corresponding derivatives **10** and **11**, respectively.

#### 4.1.12. 3-Amino-2-((2-azidoethyl)thio)-3,5,6,7-tetrahydro-4H-cyclopenta[4,5]thieno[2,3-d]pyrimidin-4-one (10)

Yield: 75%, mp 189–190 °C. IR (KBr, *υ*, cm^−1^): 3316, 3205 (NH_2_), 2102 (N_3_), 1668 (C═O), ^1^H NMR (DMSO-d_6_, *δ* ppm), 2.17–2.23 (m, 2H, CH_2_), 2.52 (t, 2H, *J* = 2.8 Hz, CH_2_), 2.91 (t, 2H, *J* = 7.2 Hz, CH_2_), 3.15 (t, 2H, *J* = 6.8 Hz, CH_2_), 3.37 (t, 2H, *J* = 6.8 Hz CH_2_), 5.71 (br s, 2H, NH_2_ exchangeable with D_2_O). MS: *m/z* (%)= 309 (M+, +1, 14). Anal. Calcd. for C_11_H_12_N_6_OS_2_ (308.38): C, 42.84; H, 3.92; N, 27.25. Found: C, 42.70; H, 3.85; N, 27.34.

#### 4.1.13. 3-Amino-2-((4-azidobutyl)thio)-3,5,6,7-tetrahydro-4H-cyclopenta[4,5]thieno[2,3-d]pyrimidin-4-one (11)

Yield: 77%, mp 186–187 °C. IR (KBr, *υ*, cm^−1^): 3296, 3191 (NH_2_), 2101 (N_3_), 1680 (C═O); ^1^H NMR (DMSO-d_6_, *δ* ppm): 2.25–2.33 (m, 2H, CH_2_), 2.39–2.48 (m, 2H, CH_2_), 2.50 (m, 2H, CH_2_), 2.89 (t, 2H, *J*= 7.2 Hz, CH_2_), 2.91 (t, 2H, *J*= 6.9 Hz, CH_2_), 3.05 (t, 2H, *J*= 6.8 Hz, CH_2_), 3.38 (t, 2H, *J*= 6.8 Hz, CH_2_), 5.69 (br s, 2H, NH_2_ exchangeable with D_2_O). Anal. Calcd. for C_13_H_16_N_6_OS_2_ (336.43): C, 46.41; H, 4.79; N, 24.98. Found: C, 46.15; H, 4.71; N, 25.08.

#### 4.1.14. General procedure synthesis of acetylated 1,2,3-triazole glycosides (13–18)

Sodium ascorbate (0.08 g, 0.4 mmol) and few drops of DIPEA were added sequentially to a mixture of the respective propargyl sugars **12a**, **12b**, or **12c** (2 mmol) and azido thienopyrimidine derivatives 10 or 11 (2 mmol) in the solvent mixture of THF–H_2_O (2:1; 15 ml). CuSO_4_·5H_2_O (0.11 g, 0.4 mmol,) was then added and the mixture was stirred at 60 °C for (6 h) (TLC, petroleum ether–EtOAc, 3:1). Ethyl acetate (30 ml) was added followed by shaking the reaction content for 5 min then the organic layer was separated, washed with water and the solvent was evaporated. The residue was purified by column chromatography (petroleum ether–EtOAc, 4:1) to afford the triazole-O-linked glycosyl derivatives **13**–**18**.

#### 4.1.15. 3-Amino-2-(ethylylthio)-{2-[4-({[2,3,4,6-tetra-O-acetyl-β-d-glucopyranosyl] oxy}methyl)-1H-1,2,3-triazol-1-yl]ethoxy}-3,5,6,7-tetrahydro-4H-cyclopenta[4,5]thieno[2,3-d] pyrimidin-4-one (13)

Yield: 65%, mp 159–160 °C. IR (KBr, *υ*, cm^−1^): 3434 (NH_2_), 1745 (C═O), 1666 (C═O), ^1^H NMR (DMSO-d_6_, *δ* ppm): 1.93, 1.96, 1.98, 2.00 (4s, 12H, 4CH_3_), 2.36–2.47 (m, 2H, CH_2_), 2.86 (t, 2H, *J*= 1.8 Hz, CH_2_), 3.00 (t, 2H, *J*= 7.2 Hz, CH_2_), 3.29 (t, 2H, *J*= 6.8 Hz, CH_2_), 3.35 (t, 2H, *J*= 6.8 Hz CH_2_), 4.02–4.05 (m, 1H, H^5′^), 4.11 (dd, 1H, *J*= 3.3, 10.8 Hz, H^6′^), 4.64 (dd, 1H, *J*= 11.3, 3.3 Hz, H^6″^), 4.81–4.87 (m, 1H, H^4′^), 5.19 (s, 2H, CH_2_), 5.21 (dd, 1H, *J*= 8.4, 9.2 Hz, H^3′^), 5.41 (t, 1H, *J*= 9.3 Hz, H^2′^), 5.65 (br s, 2H, NH_2_ exchangeable with D_2_O), 5.71 (d, 1H, *J*= 9.8 Hz, H^1′^), 8.27 (s, 1H, triazole-H); ^13^C NMR (DMSO-d_6_, *δ* ppm): 20.70, 20.81, 20.98, 21.06, 21.09, 21.14, 21.22, 21.48, 56.32, 68.50, 68.92, 69.80, 71.05, 71.21, 72.42, 99.03, 115.73, 124.63, 129.11, 139.41, 143.33, 169.77, 169.96, 170.22; 170.48, 170.53, 170.74, 172.44. Anal. Calcd. for C_28_H_34_N_6_O_11_S_2_ (694.73):C, 48.41; H, 4.93; N, 12.10. Found: C, 48.28; H, 4.83; N, 12.19.

#### 4.1.16. 3-Amino-2-(ethylthio)-{2-[4-({[2,3,4,6-tetra-O-acetyl-β-d-galactopyranosyl]oxy}methyl)-1H-1,2,3-triazol-1-yl]ethoxy}-3,5,6,7-tetrahydro-4H-cyclopenta[4,5]thieno[2,3-d]pyrimidin-4-one (14)

Yield: 72%, mp 156–157 °C. IR (KBr, *υ*, cm^−1^): 3429 (NH_2_), 1742 (C═O), 1665 (C═O), ^1^H NMR (DMSO-d_6_, *δ* ppm): 1.95, 1.96, 1.97, 2.01 (4s, 12H, 4CH_3_), 2.33–2.38 (m, 2H, CH_2_), 2.47 (t, 2H, *J*= 2.8 Hz, CH_2_), 2.90 (t, 2H, *J*= 7.2 Hz, CH_2_), 3.30 (t, 2H, *J*= 6.9 Hz, CH_2_), 3.35 (t, 2H, *J*= 6.6 Hz, CH_2_), 3.97–4.14 (m, 1H, H^5′^), 4.26 (dd, 1H, *J*= 3.3, 10.8 Hz, H^6′^), 4.29 (dd, 1H, *J*= 11.3, 3.3 Hz, H^6″^), 4.50–4.82 (m, 1H, H^4′^), 4.94 (s, 2H, CH_2_), 5.13 (dd, 1H, *J*= 8.4, 9.2 Hz, H_3′_), 5.50 (t, 1H, *J*= 9.30 Hz, H^2′^), 5.60 (br s, 2H, NH_2_ exchangeable with D_2_O), 6.20 (d, 1H, *J*= 9.8 Hz, H^1′^), 8.26 (s, 1H, triazole-H), ^13^C NMR (DMSO-d_6_, *δ* ppm): 20.87, 20.95, 21.11, 21.49, 25.68, 27.69, 29.06, 29.40, 56.10, 68.96, 69.05, 70.87, 71.02, 71.58, 71.74, 100.96, 107.29, 124.55, 136.34, 139.41, 143.42, 157.63, 162.00, 166.00; 169.44, 169.94, 170.00, 172.44. Anal. Calcd. for C_28_H_34_N_6_O_11_S_2_ (694.73): C, 48.41; H, 4.93; N, 12.10. Found: C, 48.19; H, 4.95; N, 11.98.

#### 4.1.17. 3-Amino-2-(ethylthio)-{2-[4-({[2,3,4-tri-O-acetyl-β-d-xylopyranosyl]oxy}methyl)-1H-1,2,3-triazol-1-yl]ethoxy}-3,5,6,7-tetrahydro-4H-cyclopenta[4,5]thieno[2,3-d]pyrimidin-4-one (15)

Yield: 71%, mp 153–154 °C. IR (KBr, *υ*, cm^−1^): 3441 (NH_2_), 1746 (C═O), 1669 (C═O), ^1^H NMR (DMSO-d_6_, *δ* ppm): 1.96, 1.97, 1.99 (3s, 9H, 3CH_3_), 2.34–2.48 (m, 2H, CH_2_), 2.87 (t, 2H, *J*= 2.8 Hz, CH_2_), 3.01 (t, 2H, *J*= 7.2 Hz, CH_2_), 3.31 (t, 2H, *J*= 6.9 Hz, CH_2_), 3.36 (t, 2H, *J*= 6.6 Hz, CH_2_), 4.11 (dd, 1H, *J*= 3.3, 10.8, Hz, H^5″^), 4.17 (dd, 1H, *J*= 11.3, 3.3 Hz, H^5″^), 4.40–4.58 (m, 1H, H^4′^), 4.71 (s, 2H, CH_2_), 4.87 (dd, 1H, *J*= 8.4, 9.2 Hz, H^3′^), 5.23 (t, 1H, *J*= 9.3 Hz, H^2′^), 5.68 (br s, 2H, NH_2_ exchangeable with D_2_O), 5.69 (d, 1H, *J*= 9.8 Hz, H^1′^), 8.30 (s, 1H, triazole-H). Anal. Calcd. for C_25_H_30_N_6_O_9_S_2_ (622.67): C, 48.22; H, 4.86; N, 13.50. Found: C, 48.05; H, 4.92; N, 13.36.

#### 4.1.18. 3-Amino-2-(butylthio)-{2-[4-({[2,3,4,6-tetra-O-acetyl-β-d-glucopyranosyl] oxy}methyl)-1H-1,2,3-triazol-1-yl]ethoxy}-3,5,6,7-tetrahydro-4H-cyclopenta[4,5]thieno-[2,3-d]pyrimidin-4-one (16)

Yield: 68%, mp 163–164 °C. IR (KBr, *υ*, cm^−1^): 3442 (NH_2_), 1749 (C═O), 1667 (C═O), ^1^H NMR (DMSO-d_6_, *δ* ppm): 1.53–1.58 (m, 2H, CH_2_), 1.93, 1.95, 1.97, 1.99 (4s,12H, 4CH_3_), 2.15–2.42 (m, 2H, CH_2_), 2.48 (t, 2H, *J*= 1.8 Hz, CH_2_), 2.74 (t, 2H, *J*= 7.2 Hz, CH_2_), 2.84 (t, 2H, *J*= 6.9 Hz, CH_2_), 3.16 (t, 2H, *J*= 6.9 Hz, CH_2_), 3.35 (t, 2H, *J*= 6.6 Hz CH_2_), 3.93 (m, 1H, H^5′^), 4.11 (dd, 1H, *J*= 3.3, 10.8, Hz, H^6′^), 4.14 (dd, 1H, *J*= 11.3, 3.3 Hz, H^6″^), 4.64–4.68 (m, 1H, H^4′^), 4.70 (s, 2H, CH_2_), 4.89 (dd, 1H, *J*= 8.4, 9.20 Hz, H^3′^), 5.33 (t, 1H, *J*= 9.30 Hz, H^2′^), 5.65 (br s, 2H, NH_2_ exchangeable with D_2_O), 5.68 (d, 1H, *J*= 9.8 Hz, H^1′^), 8.25 (s, 1H, triazole-H), ^13^C NMR (DMSO-d_6_, *δ* ppm): 20.28, 20.69, 20.82, 20.89, 26.05, 27.00, 27.72, 29.06, 29.51, 31.98, 56.67, 66.57, 67.98, 68.20, 70.47, 72.28, 72.42, 99.22, 115.99, 123.01, 136.67, 139.42, 144.01, 157.64, 166.47, 169.69, 169.80, 169.98, 170.43, 170.47. Anal. Calcd. for C_30_H_38_N6O_11_S_2_ (722.79): C, 49.85; H, 5.30; N, 11.63. Found: C, 49.59; H, 5.37; N, 11.52.

#### 4.1.19. 3-Amino-2-(butylthio)-{2-[4-({[2,3,4,6-tetra-O-acetyl-β-d-galactopyranosyl] oxy}methyl)-1H-1,2,3-triazol-1-yl]ethoxy}-3,5,6,7-tetrahydro-4H-cyclopenta[4,5]thieno[2,3-d]pyrimidin-4-one (17)

Yield: 70%, mp 159–160 °C. IR (KBr, *υ*, cm^−1^): 3433 (NH_2_), 1751 (C═O), 1666 (C═O), ^1^H NMR (DMSO-d_6_, *δ* ppm): 1.61–1.66 (m, 2H, CH_2_), 1.98, 1.99, 2.00, 2.01 (4s,12H, 4CH_3_), 2.39–2.48 (m, 2H, CH_2_), 2.50 (t, 2H, *J*= 1.8 Hz, CH_2_), 2.83 (t, 2H, *J*= 7.2 Hz, CH_2_), 2.94 (t, 2H, *J*= 6.9 Hz, CH_2_), 3.01 (t, 2H, *J*= 6.9 Hz, CH_2_), 3.36 (t, 2H, *J* = 6.6 Hz, CH_2_), 4.11 (m, 1H, H^5′^), 4.15 (dd, 1H, *J*= 3.3, 10.8, Hz, H^6′^), 4.19 (dd, 1H, *J*= 11.3, 3.3 Hz, H^6″^), 4.52–4.54 (m, 1H, H^4′^), 4.62 (s, 2H, CH_2_), 4.74 (dd, 1H, *J*= 8.4, 9.2 Hz, H^3′^), 5.25 (t, 1H, *J*= 9.3 Hz, H^2′^), 5.67 (br s, 2H, NH_2_ exchangeable with D_2_O), 5.70 (d, *J*= 9.8 Hz, 1H, H^1′^), 8.29 (s, 1H, triazole-H). Anal. Calcd. for C_30_H_38_N_6_O_11_S_2_ (722.79): C, 49.85; H, 5.30; N, 11.63. Found: C, 49.69; H, 5.35; N, 11.50.

#### 4.1.20. 3-Amino-2-(butylthio)-{2-{2-[4-({[2,3,4-tri-O-acetyl-β-d-xylopyranosyl]oxy}methyl)-1H-1,2,3-triazol-1-yl]ethoxy}-3,5,6,7-tetrahydro-4H-cyclopenta[4,5]thieno[2,3-d]pyrimidin-4-one (18)

Yield: 75%, mp 159–160 °C. IR (KBr, *υ*, cm^−1^), 3429 (NH_2_), 1750 (C═O), 1668 (C═O), ^1^H NMR (DMSO-d_6_, *δ* ppm): 1.62–1.64 (m, 2H, CH_2_), 1.93, 1.95, 1.99 (3s, 9H, 3CH_3_), 2.35–2.42 (m, 2H, CH_2_), 2.48 (t, 2H, *J*= 1.8 Hz, CH_2_), 2.85 (t, 2H, *J*= 7.2 Hz, CH_2_), 2.87 (t, 2H, *J*= 6.9 Hz, CH_2_), 3.01 (t, 2H, *J*= 6.9 Hz, CH_2_), 3.37 (t, 2H, *J*= 6.6 Hz, CH_2_), 4.20 (dd, 1H, *J*= 3.3, 10.8, Hz, H^5″^), 4.38 (dd, 1H, *J*= 11.3, 3.3 Hz, H^5″^), 4.60 (m, 1H, H^4′^), 4.72 (s, 2H, CH_2_), 4.88 (dd, 1H, *J*= 8.4, 9.2 Hz, H^3′^), 5.13 (t, 1H, *J*= 9.30 Hz, H^2′^), 5.67 (br s, 2H, NH_2_ exchangeable with D_2_O), 5.81 (d, 1H, *J*= 9.80 Hz, H^1′^), 8.09 (s, 1H, triazole-H), ^13^C NMR (DMSO-d_6_, *δ* ppm): 23.69, 25.68, 27.68, 28.80, 29.06, 29.40, 29.51, 30.24, 30.28, 49.31, 68.56, 68.92, 71.07, 71.29, 72.49, 99.03, 115.73, 124.63, 129.11, 139.41, 143.33, 157.63, 160.05, 169.43; 169.72, 169.96, 171.51. Anal. Calcd. for C_27_H_34_N_6_O_9_S_2_ (650.72): C, 49.84; H, 5.27; N, 12.92. Found: C, 49.72; H, 5.20; N, 13.02.

### 4.2. *In vitro* cytotoxic activity

HCT-116 (human colorectal carcinoma) and MCF-7 (human breast adenocarcinoma) cell lines were purchased from the American Type Culture Collection (Rockville, MD) and maintained in Dulbecco’s Modified Eagle’s Medium (DMEM) supplemented with 10% heat-inactivated foetal bovine serum (FBS), 100 U ml^−1^ penicillin, and 100 U ml^−1^ streptomycin. The cells were grown at 37 °C in a humidified atmosphere of 5% CO_2_.

Cytotoxicity activity against HCT-116 and MCF-7 was estimated by the 3-[4,5-dimethyl-2-thiazolyl)-2,5-diphenyl-2*H*-tetrazolium bromide (MTT) assay. This test is based on MTT cleavage by mitochondrial dehydrogenases from viable cells^43^^,^^44^. Cells were placed in a 96-well sterile microplate (5 × 10^4^ cells well^−1^) and incubated at 37 °C in serum-free media containing dimethyl sulphoxide (DMSO) and either a series of various concentrations of each compound or doxorubicin (positive control) for 48 h before the MTT assay. After incubation, the media were removed and 40 µl MTT (2.5 mg ml^−1^) was added to each well. Incubation was resumed for an additional 4 h. The purple formazan dye crystals were solubilised with 200 µl DMSO. Absorbance was measured at 590 nm in a Spectra Max Paradigm Multi-Mode microplate reader (Molecular Devices, LLC, San Jose, CA). Relative cell viability was expressed as the mean percentage of viable cells compared to the untreated control cells.

All experiments were conducted in triplicate and repeated on three different days. All values were reported as mean ± SD. The IC_50_ values were determined by SPSS Inc. probit analysis (IBM Corp., Armonk, NY).

### 4.3. Kinase inhibitory activity

The *in vitro* enzyme inhibition assessment was carried out in confirmatory diagnostic unit, Vacsera (Giza, Egypt). The evaluation performed profiling of the tested compounds against mutant EGFR kinase type L858R using gefitinib as a reference and more details were provided in the supplementary data. The *in vitro* enzyme inhibition assessment was carried out in confirmatory diagnostic unit, Vacsera (Giza, Egypt). The master mixture (6 μl 5× kinase buffer + 1 μl ATP (500 μM)+1 μl 50× PTK substrate + 17 μl water) was prepared then, 25 μl to every well was added. Five microlitres of inhibitor solution of each well labelled as “Test Inhibitor” was added. However, for the “Positive Control” and “Blank”, 5 μl of the same solution without inhibitor (inhibitor buffer) was added. Three millilitres of 1× kinase buffer by mixing 600 μl of 5× kinase buffer with 2400 μl water was prepared. Hence, 3 ml of 1× kinase buffer became sufficient for 100 reactions. To the wells designated as “Blank”, 20 μl of 1× kinase buffer was added. EGFR enzyme on ice was thawed. Upon first thaw, briefly the tube containing enzyme was spun to recover full content of the tube. The amount of EGFR required for the assay and dilute enzyme to 1 ng/μl with 1× kinase buffer was calculated. Moreover, the remaining undiluted enzyme in aliquots was stored at –80 °C. The reaction was initiated by adding 20 μl of diluted EGFR enzyme to the wells designated “Positive Control” and “Test Inhibitor Control”, after that it was incubated at 30 °C for 40 min. After the 40 minutes’ reaction, 50 μl of Kinase-Glo Max reagent was added to each well and the plate was covered with aluminium foil and incubated at room temperature for 15 min. Luminescence was measured using the microplate reader.

### 4.4. Molecular docking study

Docking analysis was performed using molecular operating environment (MOE, 10.2008) software according to the previously reported method^42^^,^^45^. The three-dimensional X-ray structure of EGFR (PDB code: 3UG2)^46^ was obtained from the Protein Data Bank through the Internet. The structure of the EGFR enzyme was prepared using Protonate 3D protocol in MOE with the default options. The co-crystallised ligand, gefitinib was used to define the active site for molecular docking. First, the co-crystallised inhibitor was re-docked into the assigned active EGFR enzyme and the root-mean-square deviation value was evaluated. Then, the molecular docking procedure was done for the newly synthesised compounds **2**, **5**, **7**, and **13**–**18** into ATP binding site of EGFR. The docking protocol was done using Triangle Matcher placement method and London dG scoring function. All minimisations were achieved with MOE until an RMSD gradient of 0.05 kcal.mol^−1^ Å^–1^ with MMFF94x force field and the partial charges were automatically calculated.

## Supplementary Material

Supplemental Material
