# Supplementary material for: Click chemistry based synthesis, cytotoxic activity and molecular docking of novel triazole-thienopyrimidine hybrid glycosides targeting EGFR
Source: J Enzyme Inhib Med Chem. 2021 Jan 28;36(1):504–16. doi: 10.1080/14756366.2020.1871335 (PMC8759726; doi:10.1080/14756366.2020.1871335)
Supplement: Supplemental Material [file IENZ_A_1871335_SM7165.pdf]

# Click Chemistry based Synthesis, Cytotoxic Activity and Molecular Docking of Novel Triazole-Thienopyrimidine Hybrid Glycosides Targeting EGFR

## Supplementary File

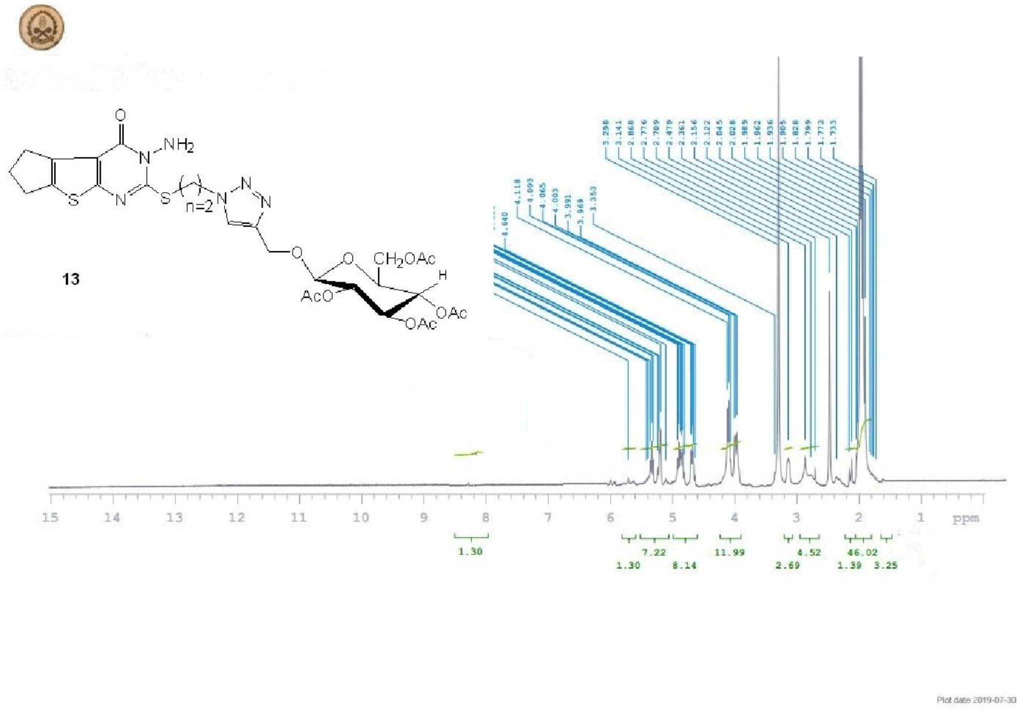


Fig 1: $^{1}$HNMR of Compound 13

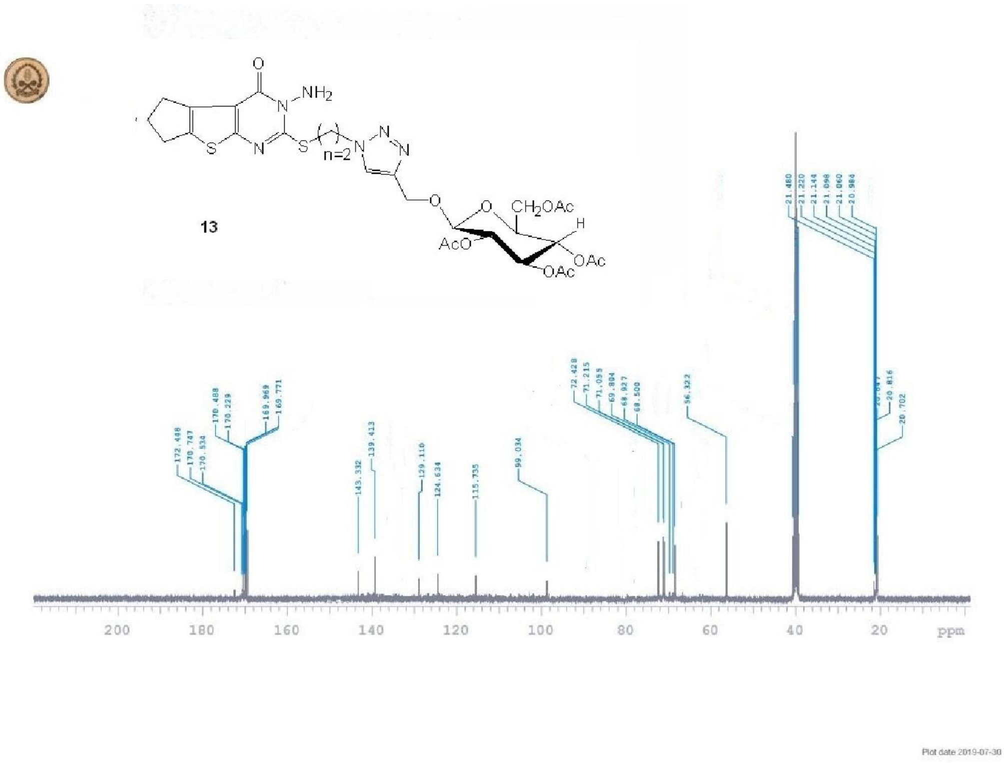


Fig 2: $^{13}$CNMR of Compound **13**

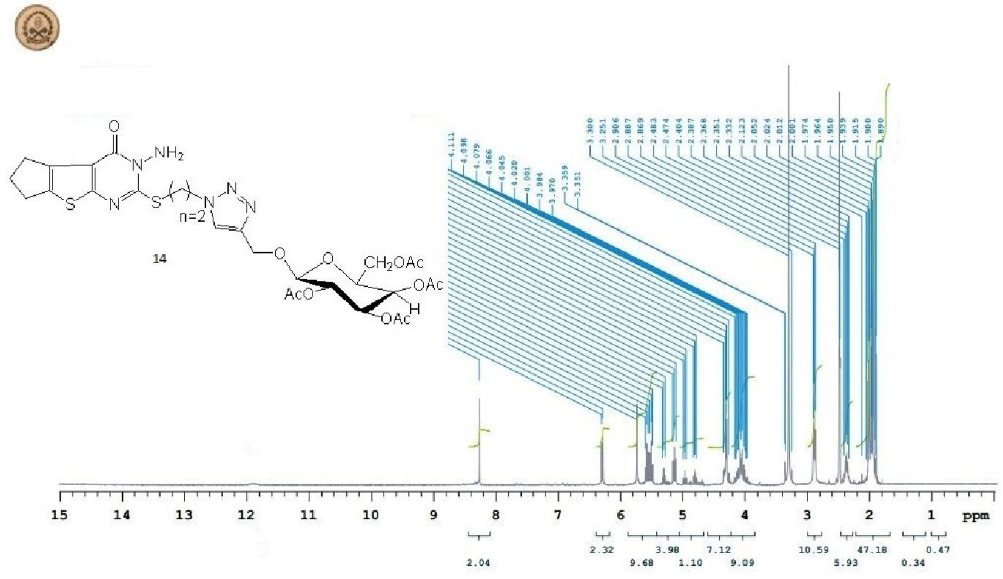


Fig 3: $^{1}$HNMR of Compound **14**

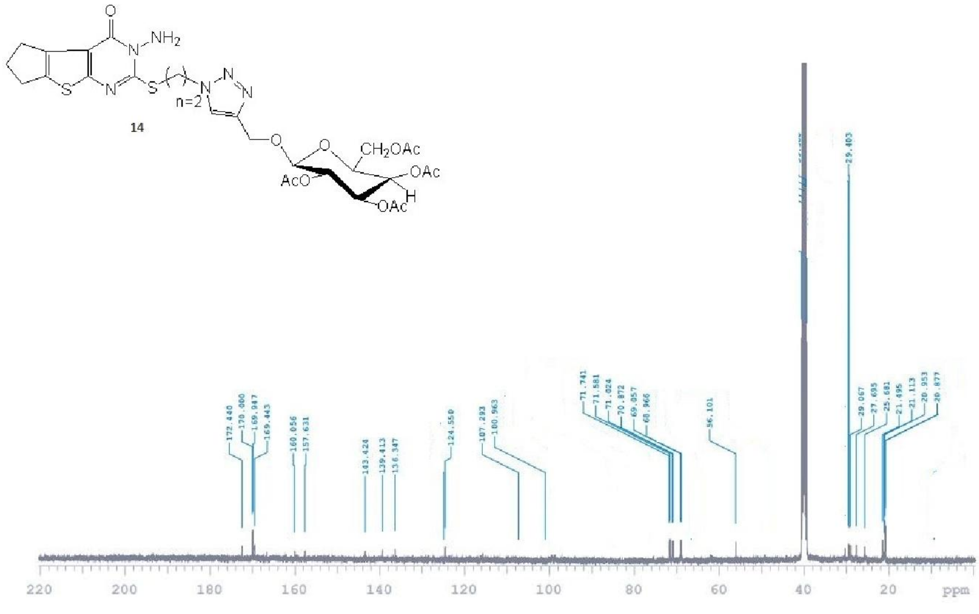


Plot date 2019-07-30

Fig 4: $^{13}$CNMR of Compound **14**

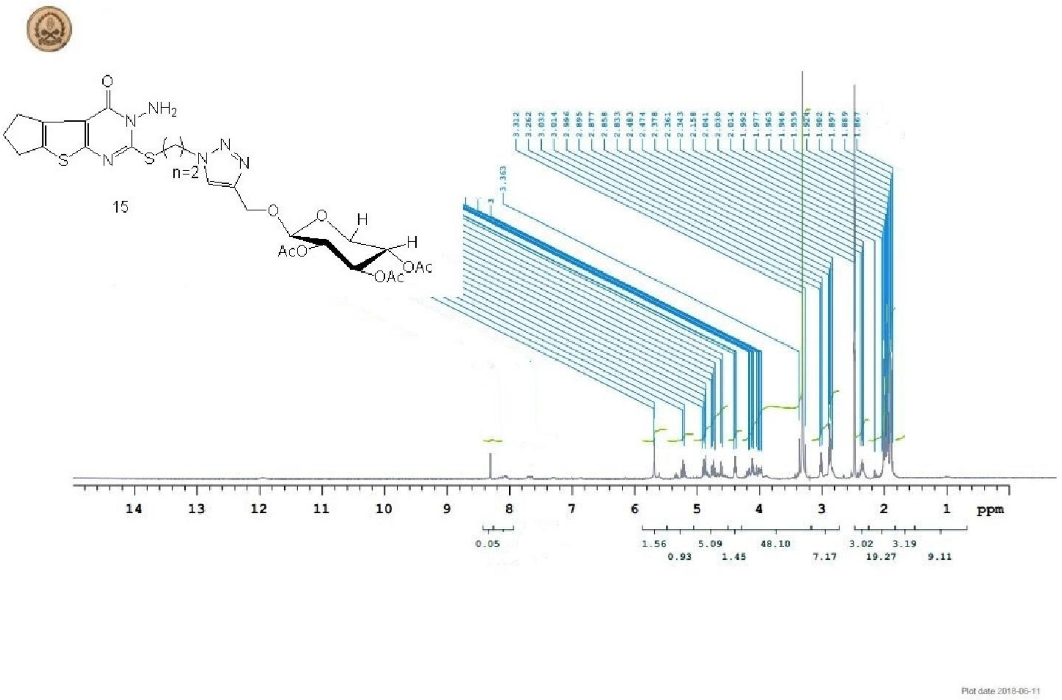


Fig 5: $^{1}$HNMR of Compound **15**

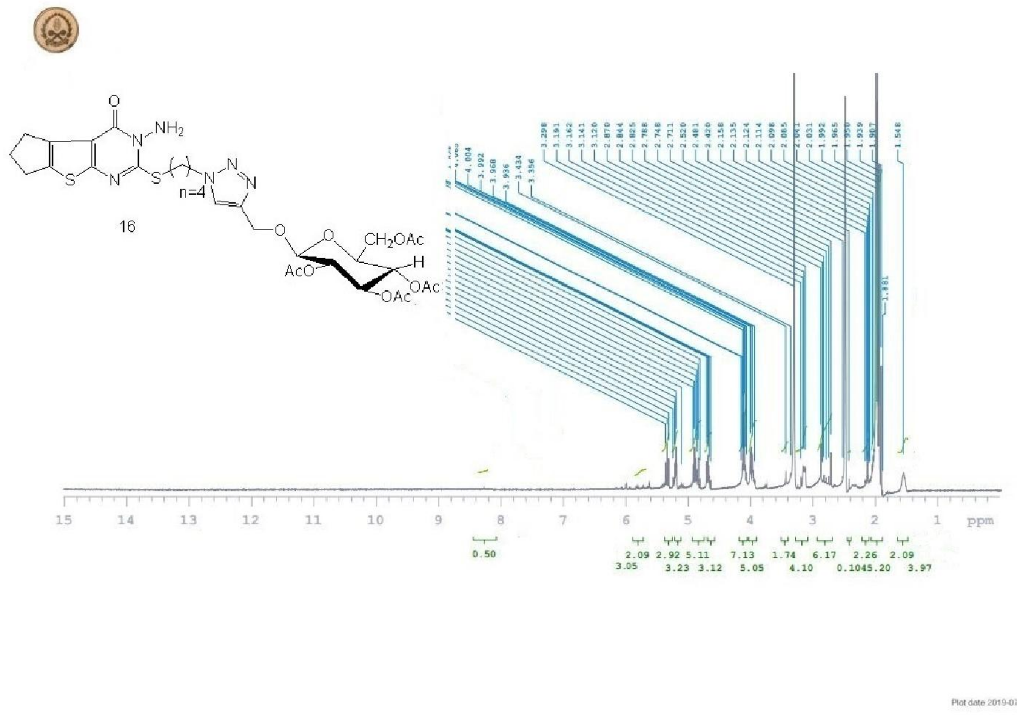


Fig 6: $^{1}$HNMR of Compound **16**

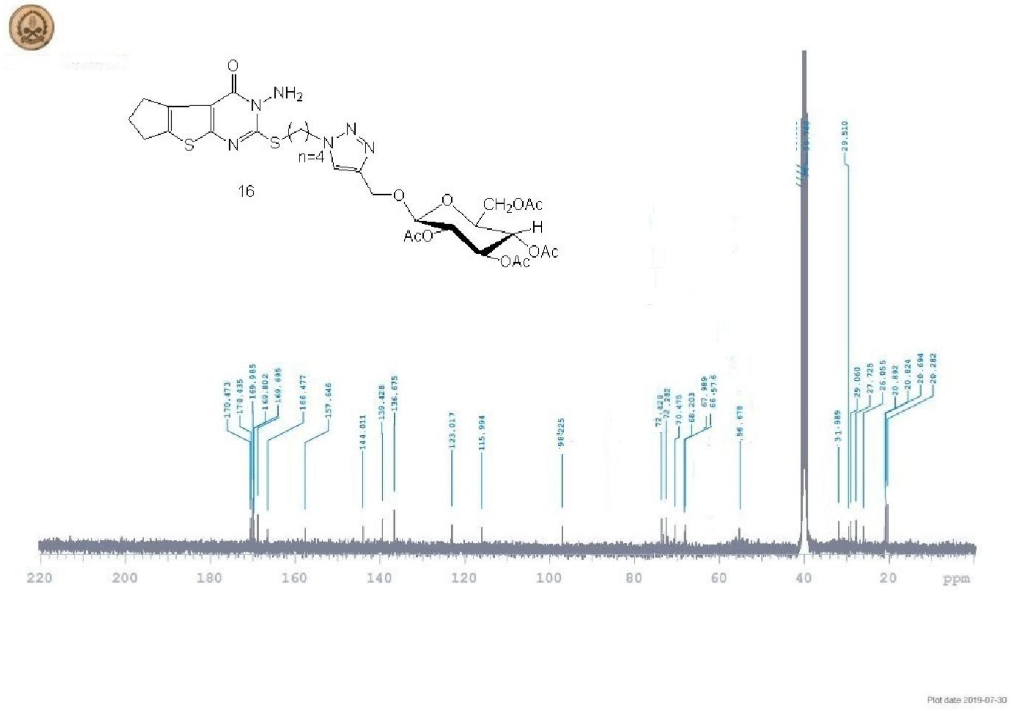

Fig 7: $^{13}$CNMR of Compound **16**

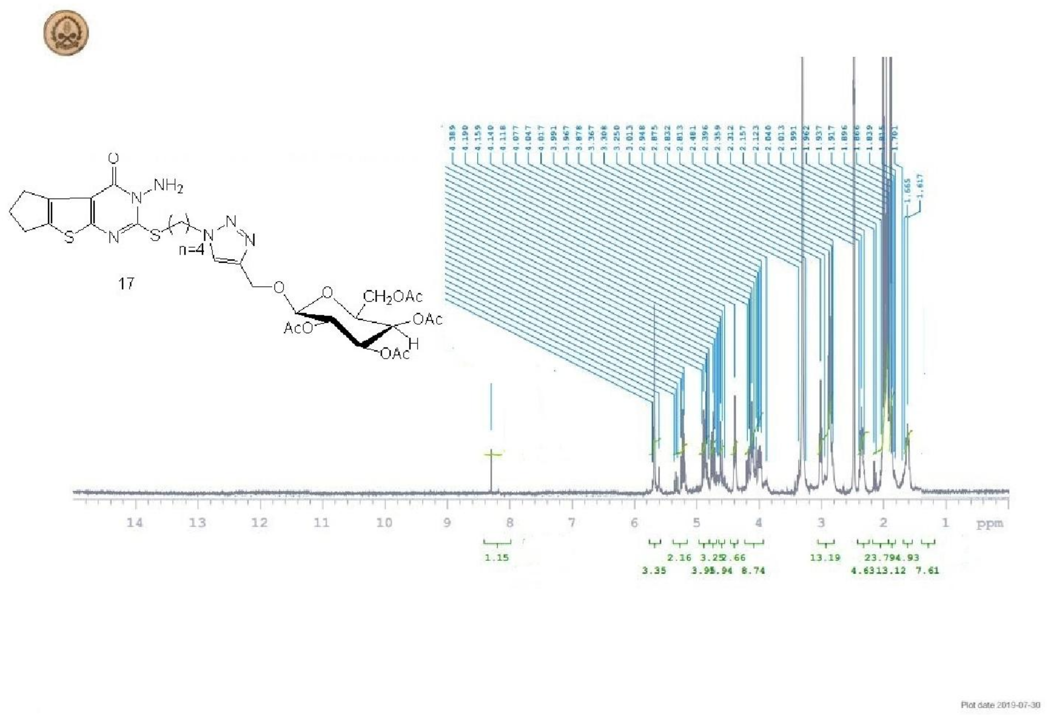

Fig 8: $^{1}$HNMR of Compound **17**

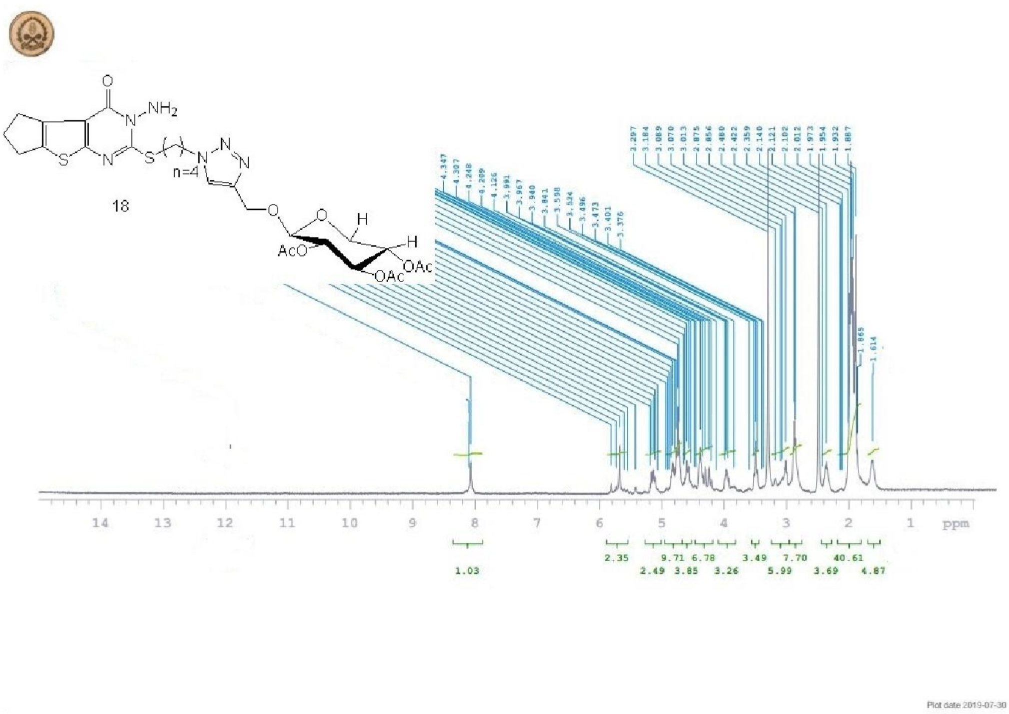


Fig 9: $^{1}$HNMR of Compound **18**

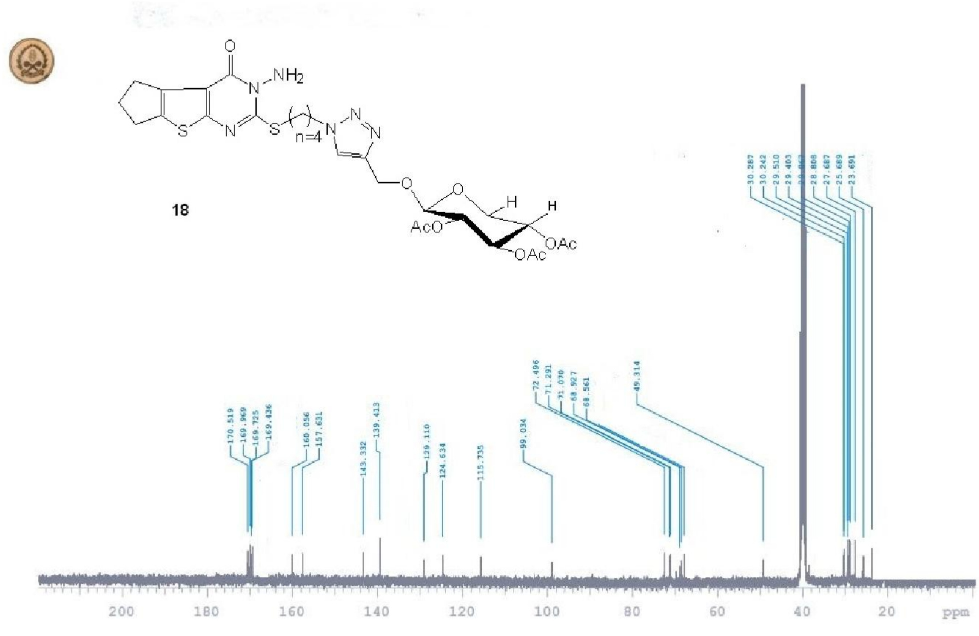


Fig 10: $^{13}$CNMR of Compound **18**
